# Patient specific real-time PCR in precision medicine – Validation of IG/TR based MRD assessment in lymphoid leukemia

**DOI:** 10.3389/fonc.2022.1111209

**Published:** 2023-01-16

**Authors:** Anke Schilhabel, Monika Szczepanowski, Ellen J. van Gastel-Mol, Janina Schillalies, Jill Ray, Doris Kim, Michaela Nováková, Isabel Dombrink, Vincent H. J. van der Velden, Sebastian Boettcher, Monika Brüggemann, Michael Kneba, Jacques J. M. van Dongen, Anton W. Langerak, Matthias Ritgen

**Affiliations:** ^1^ Hämatologie Labor Kiel, Medical Department II, Hematology and Oncology, University Medical Center Schleswig-Holstein, Kiel, Germany; ^2^ Laboratory Medical Immunology, Department of Immunology, Erasmus Medical Center (MC), University Medical Center Rotterdam, Rotterdam, Netherlands; ^3^ Oncology Biomarker Development, Genentech, Inc., South San Francisco, CA, United States; ^4^ Childhood Leukemia Investigation Prague (CLIP)-Department of Pediatric Hematology and Oncology, Second Medical Faculty, Charles University and University Hospital Motol, Prague, Czechia; ^5^ Department of Medicine III Hematology, Oncology and Palliative Care, University Hospital, Rostock, Germany; ^6^ Department of Immunohematology and Blood Transfusion, Leiden University Medical Center, Leiden, Netherlands

**Keywords:** MRD, RQ-PCR, IG rearrangement, TR rearrangement, personalized diagnostics, IVDR, method validation, EuroMRD

## Abstract

Detection of patient- and tumor-specific clonally rearranged immune receptor genes using real-time quantitative (RQ)-PCR is an accepted method in the field of precision medicine for hematologic malignancies. As individual primers are needed for each patient and leukemic clone, establishing performance specifications for the method faces unique challenges. Results for series of diagnostic assays for CLL and ALL patients demonstrate that the analytic performance of the method is not dependent on patients’ disease characteristics. The calibration range is linear between 10^-1^ and 10^-5^ for 90% of all assays. The detection limit of the current standardized approach is between 1.8 and 4.8 cells among 100,000 leukocytes. RQ-PCR has about 90% overall agreement to flow cytometry and next generation sequencing as orthogonal methods. Accuracy and precision across different labs, and above and below the clinically applied cutoffs for minimal/measurable residual disease (MRD) demonstrate the robustness of the technique. The here reported comprehensive, IVD-guided analytical validation provides evidence that the personalized diagnostic methodology generates robust, reproducible and specific MRD data when standardized protocols for data generation and evaluation are used. Our approach may also serve as a guiding example of how to accomplish analytical validation of personalized in-house diagnostics under the European IVD Regulation.

## 1 Introduction

Allele-specific oligonucleotide real-time quantitative PCR (ASO-PCR) is an accepted method in the field of specialized diagnostics for hematologic malignancies to analyze minimal residual disease (MRD). It is being used for several decades, has been established as a prognostic marker (1–4) and clinical standard in many different lymphatic neoplasms (5–8). The test results guide clinical decisions in the therapy of acute lymphoblastic leukemia (ALL) and highlight the influence of analytical methods in precision medicine. MRD is the term used for small numbers of malignant cells that remain in the peripheral blood (PB) or bone marrow (BM) during or after treatment. Its detection is based on the junctional region of rearranged immunoglobulin (IG) heavy and light chain genes and T-cell receptor (TR) genes, which are fingerprint-like sequences that can be used as clone-specific PCR targets for the vast majority of B- and T-cell neoplasias (9, 10). In contrast to methods that detect a common target in different samples, for each patient and each individual PCR target an allele-specific oligonucleotide (ASO-primer) is designed. The ASO primer is combined with an adequate primer/probe system to generate a specific real-time quantitative PCR assay, which detects the tumor-related junctional region (CDR3) identified by combination of multiplex PCR and nucleic acid sequencing at the time point of diagnosis or relapse.

High analytical standardization of this methodology has been reached by an international collaboration, the EuroMRD Consortium, which published a data evaluation and interpretation guideline for ASO-PCR (11). The current level of analytical standardization has been reached by the quality objectives of the EuroMRD Consortium organizing round robin testing, training meetings and carefully evaluating the results and pitfalls of the method, as well as by addressing the biology of the targeted molecules and the principles of PCR amplification (11–13). Due to the patient-specific nature of the test, the required validation evidence for a diagnostic assay has only been assessed in a limited way for ASO-PCR. Repeated analysis under different conditions using a defined number of samples to address precision, accuracy or robustness of each individual ASO-PCR assay would require a major investment in time and costs to establish such a test for each patient. Additionally, due to the lack of sufficient reference materials, medical samples for e.g. ALL will only be available by additional bone marrow aspirations from patients.

Several scientific reports from different fields (14–17), regulatory guidelines (18, 19) or standards from different sources (Clinical Laboratory Improvement Amendments (CLIA), Clinical Laboratory Standards Institute (CLSI), International Organization for Standardization (ISO)) exist, addressing the scope and amount of validation activities. There is considerable variation in the suggested scope of a validation and it is necessary to adapt them to the specific analytical method and its intended use. Nevertheless, validation of a method is a formal requirement, both in applicable international standards for the quality management of analytical laboratories, and in regulations from health authorities. It is also included in the European IVD regulation (20) as part of Article 5.5, which besides other declarations and justifications force the laboratories’ documentation to adequately address analytical and clinical performance of the used methods.

The current report summarizes retrospective and prospective analytic studies for the validation of the ASO-PCR method. It shows that despite the need for patient-specific reagents, sensitive, precise and accurate results can be obtained, even in the context of the latest developments in MRD techniques. The described approach may serve as a practical guide to laboratories, which have to adequately and reliably align their methods with regulatory requirements.

## 2 Materials and methods

**Figure 1:**
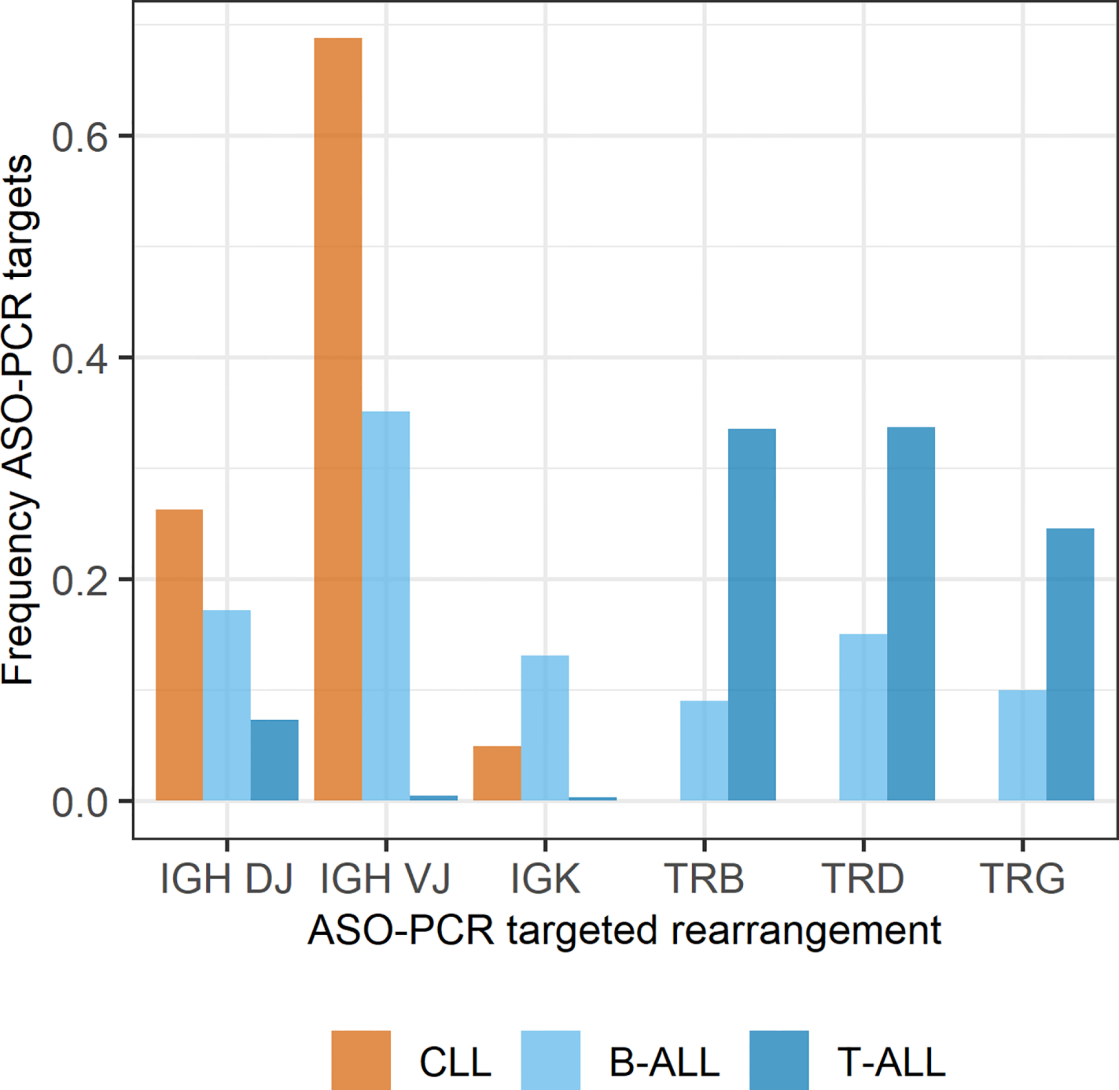
Frequency of IG and TR rearrangements as ASO-PCR targets in actively used MRD assays of patients with CLL (N=520), B-ALL (N=1078), and T-ALL (N=322). In accordance with the literature (21), 23% of the ALL patients in our assessment had a T-ALL diagnosis and 77% were diagnosed with B-ALL. For 88% of the patients two MRD assays are established, and ~10% of patients have only one assay for MRD assessment in both ALL sub-entities, thereby allowing assessments on the distribution of ASO-PCR targets from 520, 2110, and 614 assays for CLL, B-ALL and T-ALL, respectively.

The scope of the validation was defined from the frequency of the possible PCR targets evaluated from actively managed assays for B-cell chronic lymphocytic leukemia (CLL) (520 assays) and ALL patients (2110 assays B-ALL, 614 assays T-ALL, **Figure 1**). Preliminary results and further planned activities have been presented to the US Food and Drug Administration (FDA) at several meetings in the application process for ASO-PCR as a drug development tool for CLL clinical trials. After collecting the performance data for CLL, a comparable data set was selected for actively used ALL ASO-PCR assays considering the proportion of the different rearrangement types determined during the preparation of the work and B- and T-ALL frequencies (21–23).

Materials: The validation approach included analytical data from peripheral blood samples collected during the clinical trials CLL11(NCT01010061), CLL14 (NCT02242942) and bone marrow samples collected during GMALL trials 07/03 (NCT00198991), 08/13 (NCT02881086) and GMALL registry, as well as prospectively analyzed blood samples evaluated in two EuroMRD laboratories. Informed consent was obtained from all subjects involved in the study. Samples for retrospective analysis were randomly selected from a data pool that fulfilled the necessary criteria according to the EuroMRD guidelines (11).

Methods: ASO-PCR of the patient-specific and the control gene assays was performed using the BCR master and detection kit (Roche Diagnostics GmbH, Penzberg, Germany) for CLL, the Lightcycler 480 Probe Master buffer (Roche Diagnostics GmbH, Penzberg, Germany) or QPCR Mastermix Plus (Eurogentec, Seraing, Belgium) for ALL as described by Cazzaniga et al. (24). Assay results were evaluated according to the EuroMRD guidelines (11). MRD values were calculated as the ratio of tumor cells per number of analyzed nucleated cells as assessed by RQ-PCR of the albumin single copy housekeeping gene. Whenever possible the guidelines and standards from the Clinical Standards Laboratory Institute (CLSI) (25–29) were used to derive an appropriate experimental set up and the recommended statistical analysis. Two EuroMRD laboratories were involved in the validation activities. Statistical computing was performed using R (30) and R studio (31).

The considered parameters, the way these were experimentally addressed, as well as the method and standards used for statistical analysis are summarized in **Supplementary** **Table 1**. For easier reading throughout the report the term Cp will be used for results obtained by the different data evaluation techniques for RQ-PCR (Cp representing the cycle crossing point of the second derivative of the measured fluorescence intensities during the PCR amplification and Ct representing the cycle threshold set by the user at which exponential amplification during the PCR reaction starts).

**Table 1:** Accuracy of ASO-PCR at the MRD cut-off 10^-4^.

Comparator method	Entity	Median Sensitivity ASO-PCR	Median Sensitivity comparator	Samples	Patients	Overall agreement [%]	Positive agreement [%]	Negative agreement [%]
Flow cytometry*	CLL	7.90E-06	4.70E-06	2233 PB	304	92.7 (91.5/93.7)	92.7 (90.7/94.2)	92.7 (91.2/93.9)
NGS**	CLL	7.80E-06	2.70E-06	62 PB	23	75.8 (63.8/84.8)	84.1 (70.6/92.1)	55.6 (33.7/75.4)
Flow cytometry*	ALL	1.30E-05	5.00E-06	357 (104 PB, 253 BM)	137	94.1 (91.2/96.1)	94.2 (89.7/96.8)	94.0 (89.6/96.6)
NGS	ALL	4.60E-06	4.60E-06	105 (24 PB, 81 BM)	14	94.3 (88.1/97.4)	91.3 (73.2/97.6)	95.1 (88.1/98.1)

* 4-color or 8-color flow cytometry, no bulk lysis performed, sensitivity was calculated from a threshold of 20 positive events per measured number of nucleated cells.

** high number of ASO-PCR low positive samples (<1x10^-4^) included in data set. 95% confidence interval is given in parentheses.

## 3 Results

Critical performance parameters in the process of MRD assessments concerned the primer design (resulting in success and ability of specifically quantifying the rearrangement of interest), the precision, and the accuracy of the method. These were then addressed in prospective validation activities for CLL, which is characterized by the expansion of the malignant clone in peripheral blood, and thus no additional bone marrow samples from patients were required. Additional performance parameters evaluated from retrospective data were the linearity and the lower limits of the calibration range for detection and quantitation, the stability of the medical specimen and the reagents, the interference from endogenous sample ingredients that influence DNA quality or PCR amplification, and the recovery of the amount of malignant cells from a given sample when different DNA extraction methods are used.

### 3.1 Analytical specificity of ASO-PCR is influenced by non-specific amplification

Analytical specificity is defined by the primer sequence derived from the CDR3 sequence of the different IG and TR gene rearrangements. Although, specificity of a primer has to be determined by sequence comparisons to known IG or TR sequences prior to assay establishment, non-specific amplification might still occur and is addressed according to the EuroMRD criteria using the negative control from PB buffy coat DNA. Variability in non-specific amplification has been reported to depend on the type of target, the type of sample (BM or PB) and the time point during or after therapy for ALL (32). For CLL restriction of the IG receptor repertoire is well known (33), and stereotyped sequences as well as biased somatic hypermutation patterns can be a source of non-specific amplification. When analyzed with one or more non-patient-specific assay, 8.4% (7/83) of the technical replicates of 22 MRD samples of 6 CLL patients showed non-specific amplification. Applying the clinical cut-off <10^-4^ for MRD negativity in CLL, four of the replicates were recorded as MRD negative, and only the remaining three replicates were recorded as false positives due to non-specific amplification. The observed overall ASO-PCR specificity is 96.4% (confidence interval, CI: 89.9; 98.8).

### 3.2 Accuracy of ASO-PCR is high

Comparison of the accuracy of ASO-PCR to an orthogonal method is influenced by the sensitivity (cells or cell equivalents used for testing) of both compared methods. Currently, two additional methods are routinely used to assess MRD. The first method, multiparametric flow cytometry, relies on the detection of an aberrant immunophenotype on vital cells, whereas the second approach, high-throughput next generation sequencing (NGS), detects PCR amplified rearranged IG or TR genes in a patients PB or BM DNA. The accuracy of ASO-PCR was therefore determined from comparisons with both methods, flow cytometry and NGS (**Table 1**).

Compared to flow cytometry the overall qualitative agreement (OA) of ASO-PCR at a MRD cut-off 1x10^-4^ (used in CLL to score MRD negativity of patients and assign them to the MRD low risk group in survival assessments in clinical trials, and often used to attest an MRD response in ALL (34)) is about or above 90% for ALL and CLL. Additionally, the agreement for samples with positive MRD status in both methods (positive agreement) as well as the agreement for the negative samples in both methods (negative agreement) at a 1x10^-4^ cut-off is balanced at levels >90% (**Table 1**) for ALL and CLL.

For ALL the agreement of ASO-PCR to NGS (OA 94.3%) is comparable to the agreement reported by Svaton et al. (35) (80.6%) for both methods, as well as to the one observed for flow cytometry (OA 94.1%). The CLL samples show lower agreement to NGS than to flow cytometry, with only 76% OA. This potentially results from the high number of MRD low positive samples (25/62) in the CLL data set, which were all confirmed as MRD positive by the NGS method. These samples are scored as MRD negative at the applied cut-off. Additionally the sensitivity of the NGS analysis is about three times lower compared to ASO-PCR, as the MRD is derived from a single measurement from a pool of 0.25 - 1.5 million cells compared to the mean of a triplicate measurement of up to 150,000 cells for ASO-PCR.

**Figure 2:**
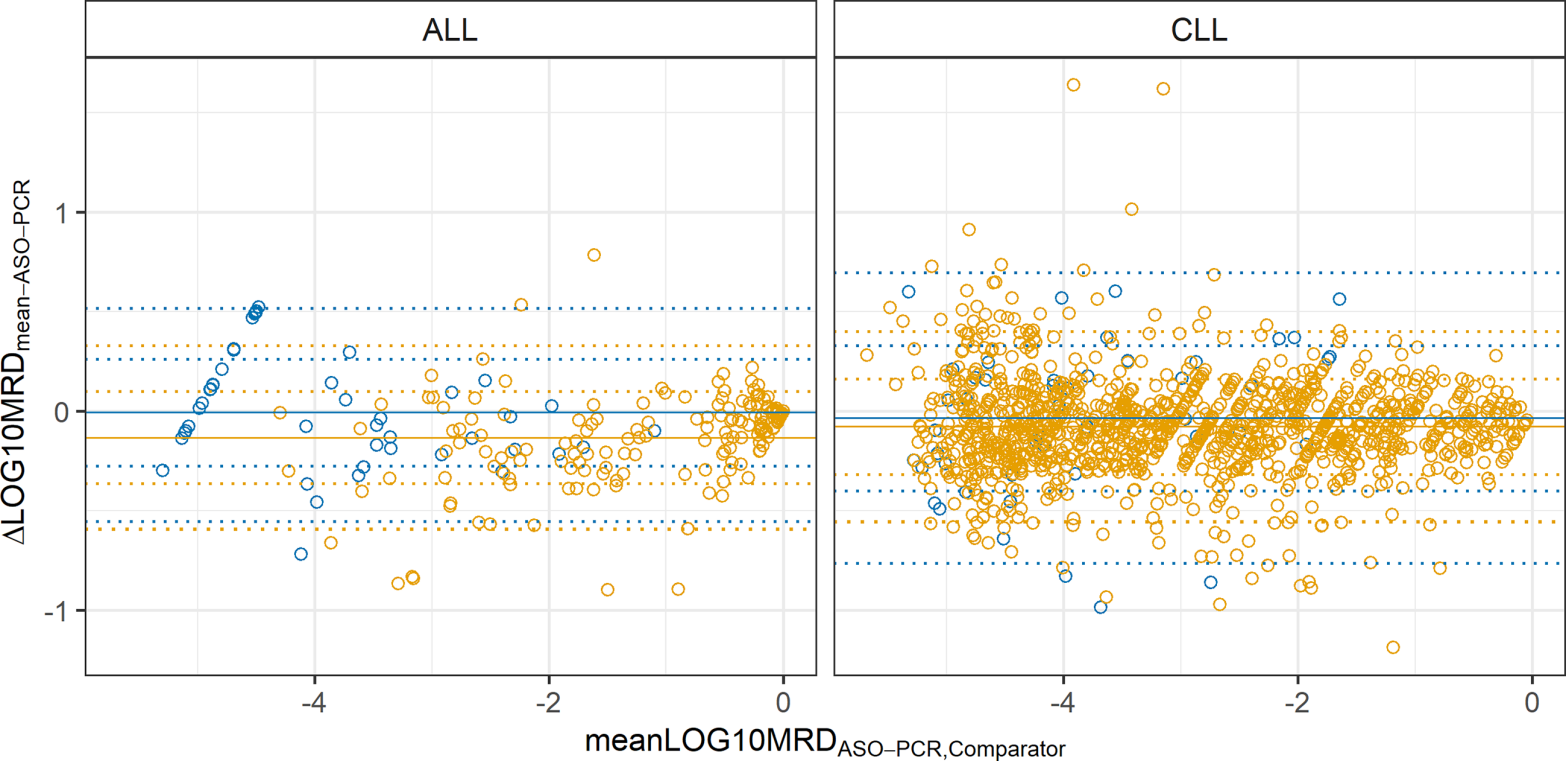
Bland-Altman plots of concordant positive MRD samples analyzed by ASO-PCR and 4-color or 8-color flow cytometry (yellow) and NGS (blue). 1205 CLL samples and 169 ALL samples were compared using flow cytometry, and 49 CLL samples and 44 ALL samples were compared using NGS. Bias (solid line) and 1S and 2S intervals (dotted lines) are shown.

For both entities and comparator methods the Bland-Altman plots show an underestimation of the MRD by ASO-PCR. For CLL this underestimation is 7.6% compared to NGS and 16.3% compared to flow cytometry (**Figure 2**, flow cytometry yellow, NGS blue). In ALL MRD is underestimated at 1.4% compared to NGS and 26.2% compared to flow cytometry. No systematic bias depending on the MRD level of the samples was detected, although the slope of the linear regressions were between 0.95 and 0.68 for CLL (R^2 =^ 0.9 and 0.64, flow and NGS, respectively) and 0.81 and 1.02 for ALL (R^2 =^ 0.86 and 0.8; flow and NGS, respectively). About 6.0% (72/1205 flow cytometry; 3/49 NGS) of the CLL samples, and 2.3% to 4.7% (1/44 NGS; 8/168 flow cytometry) of the ALL samples were outside the 2-sigma range.

An inter-lab comparison between our laboratories showed 93.5% and 86.4% OA (CLL and ALL, respectively) and details are summarized in **Table 2**. Values of positive MRD samples showed high concordance (R^2 =^ 0.94 ALL; R2 = 0.97 CLL), which underline the specificity of the different primers for a given biomarker, and the accuracy of the MRD values assessed by two different, albeit technically identical, personalized diagnostic tests.

**Table 2:** Personalized ASO-PCR assays independently developed in the two participating laboratories are highly accurate and allow quantitation of MRD samples across different laboratories.

Entity	Study design*	Samples	Patients	OA [%](CI)	PPA [%](CI)	PNA [%](CI)
ALL	EuroMRD QA task 2, 3	44	13	86.4 (73.3/93.6)	85.7 (68.5/94.3)	87.5 (64.0/96.5
CLL	equal to EuroMRD task 3	46	10	93.5 (82.5/97.8)	95.2 (84.2/98.7)	75.0 (30.0/95.4)

* The EuroMRD quality assurance program task 3 provides the biomarker sequences to the participating labs, which have to establish the ASO-PCR assay (design and test the primers, and define LOD and LOQ) before analyzing the follow-up samples. Task 2 includes the identification of the marker from the diagnostic sample. OA, overall agreement; PPA, percent positive agreement; PNA, percent negative agreement; CI 95%, confidence interval.

### 3.3 Precision of the ASO-PCR is acceptable despite multiple sources of variability

According to the currently applied EuroMRD criteria, precision of ASO-PCR is estimated from the Cp- differences observed in replicate measurements of the standards or samples. This criterion (1.5 Cp) has been derived from the variation of the reporting signals mean to the minimum and maximum reporting signals from >100 samples (10). No estimates for the variance for the calculated MRD values have been published so far. Therefore an experiment was set up to investigate the influence of different sources of variation on the MRD assessment by ASO-PCR. A spike-in approach using 3 CLL patient samples was used to evaluate the effect of random factors on the variance of the method at different MRD levels. Intermediate precision for the MRD range ≥10^-4^ was <50% at both laboratories, and increased further for MRD levels of ~10^-5^ due to the exponential character of the conversion of Cps to copy numbers (**Table 3**). Considering the data of both labs, none of the tested random factors can be determined as having an equal impact on the total variance of the method across the complete MRD range. Within-day precision (including the within-run precision) contributes evenly and significantly to the overall precision. The MRD status determined from the 144 spiked samples in the two laboratories were highly concordant with an OA of 89.2% (CI: 83.5; 93.1) using the clinical MRD negativity cut-off 10^-4^, which does not consider qualitative and quantitative RQ-PCR results at concentrations <10^-4^. The OA increased to 97.0% (CI: 93.0; 98.7) when the limit of detection was used for the definition of negativity.

**Table 3:** Precision estimates derived from a mixed effects regression model of a spike-in experiment of different patient assays (n=3) for different random factors.

nominal MRD	mean MRD	SD Lot	SD Operator	SD Day	SD Repeat	SD_total_	CV_total_ [%]
1.00E-02	1.08E-02	3.10E-07	1.93E-03	1.47E-07	3.64E-04	3.97E-03	36.79
1.00E-03	1.13E-03	6.49E-08	2.17E-04	6.56E-05	1.04E-09	5.23E-04	46.32
3.20E-04	3.58E-04	1.97E-08	6.39E-05	2.39E-05	1.10E-11	1.70E-04	47.44
1.00E-04	1.04E-04	7.12E-09	1.78E-05	1.11E-05	1.85E-11	6.33E-05	60.91
3.20E-05	4.02E-05	6.27E-09	9.02E-06	9.05E-06	3.89E-12	4.29E-05	106.8
1.00E-05	2.41E-05	4.58E-06	7.46E-06	8.92E-06	1.97E-09	4.42E-05	183.4

Assays have been performed using different reagent lots (n=2) by different operators (n=4, 2 at each laboratory), and repeated on different days (n=3) including technical replicates (n=3) per analysis to evaluate influence on the variance of MRD measurements.

Results of repeated measurements to evaluate intermediate precision for the assessment of clinical specimen can be found in the **Supplementary Material** (**Supplementary Table 2**). The precision estimate for the clinical samples are in good accordance to the precision estimates determined from the spike-in experiment, demonstrating the applicability of the spike-in approach using only a very small number of patient samples to establish a precision estimate for ASO-PCR.

### 3.4 Linearity and LOD of ASO-PCR easily reach the 10^-4^ level and even beyond

The linearity and limits of the calibration range might be affected by a number of factors that influence the amplification of the target during the PCR reaction like starting concentration, reagent quality, amplification efficiency (influenced by factors like mutation status of the target), or primer design. Linearity and limit of detection (LOD) were evaluated from a total of 90 assays (60 CLL, 30 ALL). Deviations from linearity can be visually recognized from a number of standard curves (**Supplementary Figure 1**), and statistical analysis of the linear and nonlinear models revealed significant nonlinearity for 26/60 CLL assays and 21/30 ALL assays (p<0.05). The deviation from linearity for each of the technical replicates from these assays was tested (2^nd^ and 3^rd^ order models) against the set acceptance limit of ≤1.32 Cp deviation from linearity. This acceptance limit is based on the estimated maximum Cp difference that would be observed if the slope differs at acceptable levels (EuroMRD: -3.1 to -3.9 (11),) from the theoretical slope -3.3 for a PCR amplification efficiency of 2. Thus, in total 91.9% (55/60) of CLL assays and 96.7% (29/30) of ALL assays show a linear calibration range from 10^-1^ to 10^-5^.

Due to the methodological limitations of the assays, leading to measurements without fluorescence signals in the PCR amplification of low concentrated samples and no template controls (blanks), the limit of blank (LOB) for ASO-PCR was determined non-parametrically from the measured Cps using the signal reporting limit of 45 Cp. Based on the Cp, the LOB for CLL were 45 and 40.8 for the two participating laboratories, respectively. Using a probit approach with a hit rate of 95%, where a hit is defined by Cp<LOB, the corresponding LODs were calculated at 5.1x10^-5^ and 6.3x10^-5^. For ALL the Cp for LOB was determined at 43.5, and LOD was 5.8x10^-5^.

### 3.5 Sample quality and reagent stability effects on ASO-PCR MRD measurements are minor

**Figure 3:**
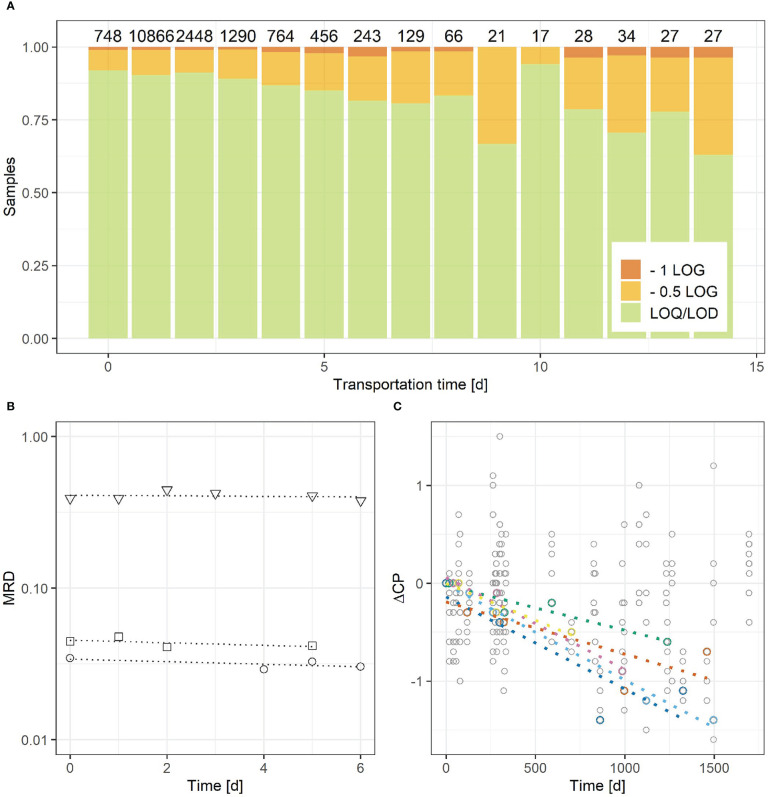
Assessment of short-term stability of medical samples **(A, B)** and long-term stability of reagents **(C)**. **(A)** Short-term stability was evaluated based on the need to adapt the analytic sensitivity of an MRD assessment due to a lower number of cell equivalents per ng of DNA in a test. If ≥50% of the required cell equivalents could be tested no adaptation of analytic performance was required (LOQ/LOD, green). LOQ and LOD is reduced by 1 LOG or 0.5 LOG level if <10% or <50% of the required cells would be tested, respectively. Cell equivalents were calculated from the copy number of the albumin gene determined by RQ-PCR according to (9). **(B)** PB was used to evaluate the potential drift of the MRD value from 3 CLL samples up to 6 days ambient storage after sample collection to prospectively assess short-term stability of medical samples. **(C)** Long-term stability was assessed from the Cp signal drift of a total of 60 primer and standard combinations from 10 CLL assays and during time periods of up to 4 years (only standards with significant Cp-drift are colored).

Usually, medical samples are processed within a short time after sample collection, whereas reagents are used over a longer time period according to the shelf life given by the manufacturer, but storage time of samples and reagents, or repeated freeze-thawing of reagents might influence the quality of MRD assessments. In a global survey to assess the short-term stability of medical samples based on the DNA quality of >17,000 samples received for MRD analysis, a significant drop in DNA quality is seen after 6-7 days after sample collection (**Figure 3A**), but 89.4% of samples are received within 3 days. Additionally, no significant MRD drift upon storage of initial whole blood samples of 3 CLL patients for up to 5 or 6 days on ambient temperature was observed (**Figure 3B**). A similar experiment with bone marrow samples from either ALL or CLL patients was not performed, as the sample amount required to provide enough material for repeated DNA extractions and subsequent analysis in general exceeds the available surplus of samples from daily routine.

Results for the short-term stability of reagents subjected to repeated freeze-thaw cycles can be found in the **Supplementary Material** (**Supplementary Figure 2**). Laboratory supplied reagents kept under quality control are not affected by freeze-thawing during short time periods and seem to be highly stable for up to at least ~3-4 years. From the standard dilution series of 10 CLL assays, 0.04% (1/264) of the repeated standard measurements in the range of 10^-1^ to 10^-4^ showed a ΔCp ≥ ± 1.5 compared to the mean Cp determined during first analysis using the same DNA dilution and the same primer stock solution (**Figure 3C**). For ALL this ratio was 1.3% (6/458) using the standards from 24 assays of 14 patients. Signal drift over time, albeit not statistically significant (p>0.05), was observed for most standards. Although 10-15% of the standards had a significant signal drift (6/60 CLL; 7/48 ALL), it was observed, that only single standards from individual assays but no complete dilution series were affected.

### 3.6 Effects of DNA extraction methods and inhibitory effects of endogenous substances on ASO-PCR MRD measurements are minor

The recovery of MRD values from clinical samples was determined from spike-in samples at MRD levels of 10^-2^, 10^-3^ and 2x10^-4^. The recovered mean MRD values were twice as high as expected (**Supplementary Table 3**, automated DNA extraction using Qiasymphony). The constant overestimation of MRD values in the samples is probably derived from a systematic error introduced by the use of frozen CLL cells for the preparation of the spike-ins, but may also be related to deviations in the flow cytometric CLL count used to prepare the spike-in samples. An underestimation of MRD has already been reported from a comparison of median MRD values obtained by flow cytometric and RQ-PCR based MRD detection (36), but dilution factors between the different spike-in levels indicate that the experimental design established the correct level differences compared to the highest CLL/PBMC ratio (nominal MRD value). Results for the influence of endogenous substances on the PCR amplification are summarized in **Supplementary Table 4**.

## 4 Discussion

ASO-PCR has so far been validated by means of ongoing round robin testing in international reference laboratories and the results of these and regular training meetings of the participating laboratories have been used by the EuroMRD Consortium to highly standardize the methodology to the best laboratory practice. The study reported here demonstrates how to use retrospective and prospective data to establish general technical performance parameters of the technique. During the preparation of the validation plan the lack of official international standards or any specific published guideline for the validation of patient-specific diagnostics was noted. Validation of a method is a formal requirement included in both the applicable international standards for the quality management of certified analytical laboratories, and the national as well as international legislations like e.g. the European IVDR (20). Neither publications by the scientific community (15, 16) nor applicable official guidelines provided satisfactory guidance to accept and qualify a patient-specific biomarker.

Additionally, because patient material is very limited for patient-specific diagnostics, it is mandatory for clinically relevant diagnostics like ASO-PCR to conclude on the type of experiments and number of samples or patients needed at an early time point in the validation process. The general performance parameters of ASO-PCR could be established using FDA recommendations resulting from the pro-active dialog between the authors and health authorities. Although, only runs previously accepted according to the EuroMRD criteria were included in the data set, the results showed that the validation method and the acceptance criteria for ASO-PCR evaluation as developed from the technical understanding of the methodology and the EuroMRD harmonization efforts (10, 11) are equally effective for the assessment of the assay parameters. Whereas EuroMRD criteria are applied to an individual assay, the results described here enabled to establish the general performance of this diagnostic method across different patients, entities and clinical time points. Still, there are parameters that need to be evaluated and validated on an individual patient’s basis, like the primer specificity and specific LOD. Inter- and intra-patient variability has also been discussed as limitation for the molecular gene fusion *BCR::ABL* as a surrogate endpoint in clinical trials of CML (37). As no patient specific reagents must be provided for the detection of *BCR::ABL*, this method performs at a different level than ASO-PCR. The FDA premarket clearance of a MRD detection kit for CML in 2016 pushed the method from a laboratory developed test to a standardized IVD kit. The validation of ASO-PCR to IVD standards has not been the focus of this work, but results reported here may be used to adapt the routine evaluation practice for ASO-PCR. E.g. acceptance or rejection of standards or samples which is currently evaluated by the Cp differences of the technical replicates could be assessed based on the precision of the individual standard or sample measurements as in common procedures of clinical biochemistry (38).

One limitation of our study is that even though we did address the effect of reagent stability on MRD measurements, it has not been possible to precisely evaluate the influence of long term storage of the laboratory supplied reagents using identical reagent lots for the time span needed for a clinical study. Shelf life and turnover times of purchased reagents were too short to cover repeated analysis within 3 years using the same reagent lot, and the amount of patient material was limited. The slow, although not significant increase of the Cp observed for most of the standards could result from the variability caused by the use of different reagent lots over the required time span or by other causes that affect the stability of the reagents over time such as freeze-thawing or chemical processes occurring upon prolonged storage. However, most of these causes would affect standards, samples and albumin measurements in the same way, and should therefore have only minor influence on the MRD results.

First evidence has been published showing that additional MRD scoring at concentrations <10^-4^ would show a benefit for prediction of progression free or overall survival in patients with ALL (1, 39, 40) and CLL (41, 42). Nevertheless, a difference in relapse-free survival of patients being clearly MRD negative compared to patients being MRD positive ≤10^-4^ has already been observed at early treatment time points using ASO-PCR (43, 44) and flow cytometry (2) for childhood and adult ALL. Low level positivity in ASO-PCR is strongly influenced by the data interpretation using the PCR signals of polyclonal healthy control samples. An investigation of the specificity of the reaction products from ASO-PCR of healthy control samples reported 30-40% (IG) and even up to 90% (TR) nonspecific positive signals, which can affect 10-65% of the measured samples depending on the time point the sample was collected (32). Fronkova et al. reported, that 15-20% of clinical samples can give nonspecific positive RQ-PCR signals when tested with a non-patient-specific assay (44), while NGS confirmed low-level positive ASO-PCR results in 61% of cases at treatment week 16 (40). Additionally, the definition of MRD negativity is for both reasons, specificity and sensitivity, under continuous discussion, especially for those cases, where the MRD evaluation has been implemented in the patient care and is no longer merely a research tool (12). The pros and cons of ASO-PCR and flow cytometry to assess MRD levels in CLL patients within the boundaries of clinical studies but also in clinical routine have also been extensively discussed (45–47) and the necessary regulatory requirements were included in the European Medicines Agency guideline on the evaluation of anticancer medicinal products in man effective since July 2016 (48).

As Wendtner (49) pointed out in a comment to the article by Thompson et al. (41) it needs sophisticated standards to provide reliable MRD data at the currently aimed sensitivity level of one in a million cells, independently of the investigated entity. Multicolor-flow cytometry or next generation sequencing being equally or more sensitive or less time consuming than ASO-PCR for MRD assessment, are currently either on the technical level or on the clinical level not validated and standardized to the same degree as ASO-PCR. Especially the NGS methodology which is strongly pushed to diagnostic use in the field of hematology and precision medicine (35, 41, 42, 50–52) and could be used to quantify MRD without the use of personalized standards and reagents, requires profound validation and experience from clinical trials to circumvent false negative results and to deliver valuable disease prognosis. This is also underlined by the accreditation requirements of the laboratory standard released from the College of American Pathologists for NGS-based clinical tests (53), which does not include requirements for quantitative aspects of molecular oncology and emphasizes that a consensus on practice must be built from professional experience.

In summary, our results demonstrate that comprehensive technical performance level validation according to regulatory requirements can also be achieved for the patient specific ASO-PCR approach.

## Data availability statement

The raw data supporting the conclusions of this article will be made available by the authors, without undue reservation.

## Ethics statement

The studies involving human participants were reviewed and approved by CLL11: University Cologne Ethical Review Committee, Medical Faculty University Cologne, Cologne, Germany CLL14: University Cologne Ethical Review Committee, Medical Faculty University Cologne, Cologne, Germany GMALL07/03: Goethe University Frankfurt Research Ethics Board, Medical Faculty, Johann Wolfgang Goethe-University, Frankfurt/Main, Germany GMALL08/13: Goethe University Frankfurt Research Ethics Board, Medical Faculty, Johann Wolfgang Goethe-University, Frankfurt/Main, Germany. The patients/participants provided their written informed consent to participate in this study.

## Author contributions

Research Design AS, JR, AL, JD, MR. Formal analysis, investigation and data curation AS, MS, MN, DK, AL, VV, EG-M, JS, SB, MR. Resources: MB, MK, JD. Writing - original draft preparation: AS. Writing - review and editing: ID, MS, MR, MB, SB, MK, MN, VV, AL, JD, DK, JR, EG-M, JS. All authors have read and agreed to the published version of the manuscript.
