# Supplementary material for: Patient specific real-time PCR in precision medicine – Validation of IG/TR based MRD assessment in lymphoid leukemia
Source: Front Oncol. 2023 Jan 16;12:1111209. doi: 10.3389/fonc.2022.1111209 (PMC9885152; doi:10.3389/fonc.2022.1111209)
Supplement: Supplementary file 1 [file DataSheet_1.pdf]

Supplementary Material

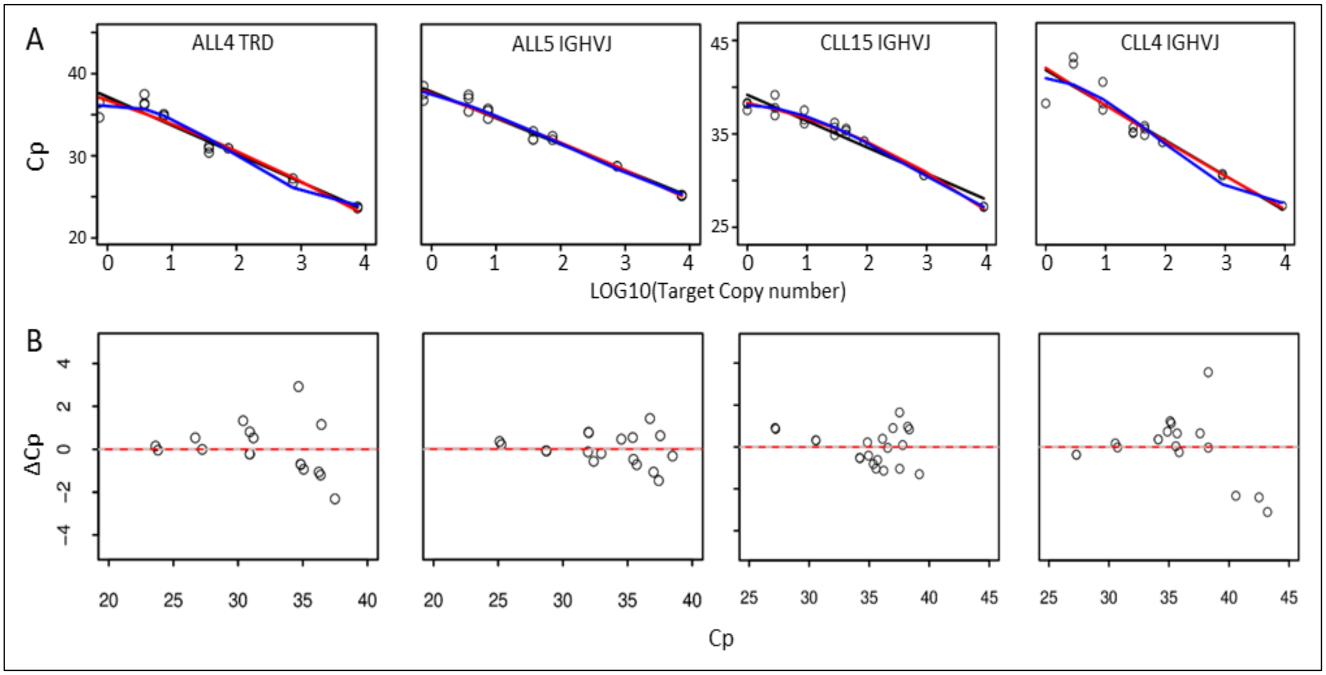

Supplementary Figure 1 Linearity of the standard dilutions of ALL and CLL ASO-PCR assays (exemplarily shown for two assays of each entity). A. Non-linear models (2nd order blue, 3rd order red) and linear model (black) of the Cp values measured for the standard dilution series. B. The distribution of ΔCp between non-linear models and linear models was tested against the ΔCp-criterion (±1.32).

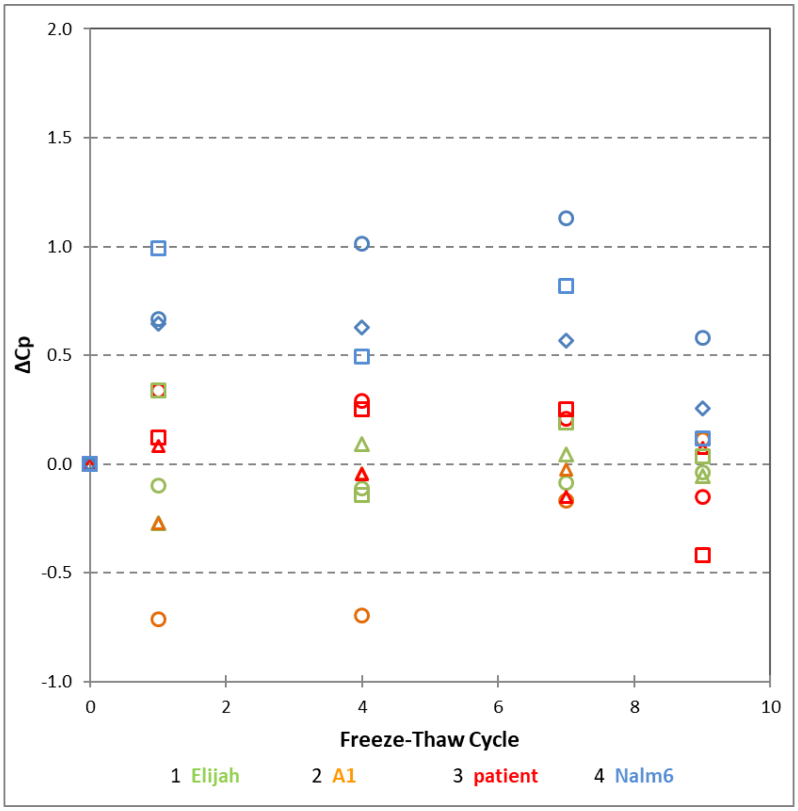

Supplementary Figure 2. Short term stability of lab supplied reagents during repeated freeze-thaw cycles. Serial dilutions of DNA from 3 cell lines (Elijah, A1, Nalm6) and 1 CLL patient were prepared and repeatedly underwent freeze-thaw cycles up to 9 times within a 4 weeks period. Aliquots from the individual freeze-thaw rounds were collected and stored frozen until all samples could be analyzed within one analytic run and compared to the value of the first cycle. With one exception ($\Delta Cp=3.75$) all measured Cp were within the 1.5 Cp criteria of the EuroMRD guideline. None of the DNA dilutions had a significant Cp drift (only concentrations covering the concentration range between 10^{-2} (circles), 10^{-3} (triangle), $5x10^{-4}$ (diamond), and of the individual assays limit of quantitation $\leq 10^{-4}$ (squares) are shown).

Supplementary Table 1. Validation approach for different performance parameter

Parameter	Standard	Objective and Method
Analytical specificity	EP12A2(26)	22 true positive samples analyzed with correct and incorrect primer
Accuracy	EP12A2(26)	358 ALL samples with flow cytometry as orthogonal method
		107 ALL samples with NGS as orthogonal method
		2233 CLL samples analyzed using flow cytometry as orthogonal method
		62 CLL samples with IG-NGS as orthogonal method
		46 follow-up samples of 10 CLL patients from individually established assays in two different laboratories
		44 ALL samples from round robin tests in the two laboratories
Precision	EP05A3(27)	Intermediate precision: multi-parametric experiment to capture sources of variation (laboratory, day, operator, reagent lot) with triplicate measurements of 6 spiked samples for MRD levels 10-2 to 10-5 using three patient specific assays analyzed using a mixed effects regression model, where the patient was defined as the basis to exert the fixed effect in the regression model, since due to genetic background, individual treatments, and sample conditions, each patient has its own intercept in the linear regression. The different operators, laboratories, days and reagent lots were used as random effects in the regression model.
		repeated analysis of 52 MRD positive follow-up samples of CLL patients
		repeated analysis of 68 MRD positive follow-up samples of ALL patients
Linearity	EP06A(28)	30 randomly chosen CLL assays
		30 randomly chosen ALL assays
LOD, LOQ	EP17A2(29)	standards from assays used for assessment of linearity

Reagent stability	EP25A(25)	standards of 8 CLL patient assays repeatedly analyzed during a period of up to four years<br><br>standards of 24 assays of 14 ALL patients repeatedly analyzed during a period of up to three years
Specimen stability	EP25A(25)	Peripheral blood samples of three CLL patients stored at ambient temperature and repeatedly extracted and analyzed during a period of 6 days
Recovery		duplicate analysis of peripheral blood samples of healthy controls spiked with CLL cells of 5 patients at three different MRD levels using 2 different DNA extraction methods
Interference		significance of differences in levels of endogenous interfering substances (hemoglobin, leukocyte count, comedication, anticoagulant, sample preparation method) determined from the proportion of samples with sufficient or insufficient DNA quality influencing ASO-PCR LOD and LOQ in 3482 peripheral blood samples and 9080 bone marrow samples as well as in a subset of 1322 peripheral blood samples of CLL patients

Supplementary Table 2 Precision of repeated analysis of medical specimen from CLL and ALL patients at different MRD levels. Mean coefficient of variation (CV) was calculated from CV obtained in the replicate measurements in the different analytic runs.

	CLL			ALL		
MRD level	n	mean CV	95% CI	n	mean CV	95% CI
>1.0E-01	11	24.74	18.1; 45.5	9	8.16	5.8;16.6
>1.0E-02	5	12.36	8.3; 58.9	12	18.0	13.3;31.9
>1.0E-03	9	16.27	11.7; 36.6	17	11.19	8.6;17.5
>3.2E-04	7	24.47	17.0; 73.0			
>1.0E-04	13	19.76	14.8; 31.2	25	12.09	9.6;17.2
≤1.0E-04	7	61.92	43.1; 184.6	5	52.29	35.0;168.0

Supplementary Table 3. Recovery of the MRD value for five individual patient assays determined from measurements of spike-in samples of tumor cells (diagnostic sample DMSO treated and stored at -80°C) to healthy peripheral blood samples at defined cell counts at ratios of 10^{-2} to 10^{-4}. The DNA of the spike-in samples was extracted using an automated (Qiasymphony, Hilden, Germany), bead based DNA extraction methodology (Qiagen, Hilden, Germany) according to the manufacturers protocol. MRD values were determined using the patient specific standard curve and recovery was calculated from the nominal MRD value set by the spike-in level.

Patient	Nominal MRD*	Albumin cell equivalents	CLL cell equivalents	Measured MRD	CV [%]	Recovery
1	0.00019	107852	36	0.00033		
2	0.00018	114921	51.3	0.00045		
3	0.00018	116046	49.3	0.00043		
4	0.00019	111129	46	0.00041		
5	0.00018	99142	35.8	0.00036		
	**0.000186**			**0.00040**	**10.0**	**2.15**
1	0.00096	107991	259	0.0024		
2	0.00090	112069	256	0.0023		
3	0.00091	116538	184	0.0016		
4	0.00095	115212	243	0.0021		
5	0.00092	83500	178	0.0022		
	**0.00093**			**0.0021**	**13.3**	**2.25**
1	0.00954	106940	2380	0.022		
2	0.00896	111690	2015	0.018		
3	0.00898	107383	1003	0.0093		
4	0.00946	111928	2190	0.02		
5	0.00912	107392	1915	0.018		
	**0.0092**			**0.017**	**25.5**	**1.9**

* nominal MRD levels were calculated using the CLL ratio determined by flow cytometry before long-term storage of the CLL samples used as spike-ins

Supplementary Table 4 Influence of endogenous sample compounds on DNA amplification of the house keeping gene albumin as a measure of RQ-PCR inhibition (A) and on the sensitivity of MRD assessments based on the number of tested cell equivalents (B). Statistical Significance was tested using the Kruskal-Wallis test. For CLL the sample time point (diagnosis DX, therapy TX, follow-up FU) was addressed in the analysis to meet concerns of high lymphocyte counts at the time point of diagnosis. (A) No significant influence of the hemoglobin content and the lymphocyte count was observed when a DNA quality criterion for the amplification of the albumin gene was used as variable. Samples below 75% of the theoretically expected value of 150 copies per ng DNA were scored as quality score failed (QSF), other samples were scored as quality score passed (QSP). QSF is not decisive for the MRD assessment of a sample and does not influence the reported sensitivity of the assay unless more or equal to 50% of the required cell equivalents can be subjected to analysis. (B) The sensitivity of the assessments could be influenced by the hemoglobin and lymphocyte content of a sample, as suggested by statistical significance. However, only 0.4% to 1.9% of samples in the individual groups did not pass the threshold for the number of cell equivalents to reach the individual assay's sensitivity. Samples were scored as sensitivity score fail (SSF, <50% cell equivalents tested) or sensitivity score passed (SSP, ≥50% cell equivalents tested).

A Quality score based on RQ-PCR amplification of albumin gene

Entity	Endogenous substance	Sample type	Quality Score	n	Median	Mean±SD	p
ALL	Hemoglobin [g l^{-1}]	BM	QSF	32	9.4	9.39±3.35	0.58
			QSP	8319	9.3	8.93±3.43	
		PB	QSF	5	8.5	9.38±2.51	0.052
			QSP	3263	11.8	11.6±2.53	
	Lymphocyte absolute count [10^9 l^{-1}]	BM	QSF	32	2.5	6.33±9.12	0.81
			QSP	8319	2.6	5.44±12.2	
		PB	QSF	5	0.8	4.38±7.51	0.72
			QSP	3263	1.2	3.39±12.3	
CLL	Lymphocyte absolute count [10^9 l^{-1}]	PB-DX	QSF	17	21.2	30.5±36.3	0.078
			QSP	519	2.9	23.9±43.6	
		PB-TX	QSF	22	0.64	0.77±0.53	0.26
			QSP	1069	0.55	0.79±1.95	
		PB-FU	QSF	57	0.8	1.62±4.07	0.19
			QSP	3147	0.7	1.08±1.73	

B Sensitivity score based on DNA amount used for MRD assessment in RQ-PCR

Entity	Endogenous substance	Sample type	Quality Score	n	Median	Mean	p
ALL	Hemoglobin [g l^{-1}]	BM	SSF	96	8.75	8.09±3.21	0.005
			SSP	8255	9.3	8.95±3.43	
		PB	SSF	21	9.9	9.97±2.2	0.002
			SSP	3247	11.8	11.6±2.53	
		BM	SSF	96	1.05	1.52±1.72	$<2x10^{-16}$

	Lymphocyte absolute count [10^9 l^{-1}]		SSP	8255	2.7	5.49±12.3	
		PB	SSF	21	0.9	1.08±0.62	0.064
			SSP	3247	1.2	3.41±12.3	
CLL	Lymphocyte absolute count [10^9 l^{-1}]	PB-DX	SSF	2	0.6	0.6±0.71	0.082
			SSP	534	3.0	24.2±43.4	
		PB-TX	SSF	7	0.7	0.76±0.42	0.45
			SSP	1087	0.56	0.79±1.94	
		PB-FU	SSF	32	0.9	1.66±3.36	0.006
			SSP	3172	0.7	1.08±1.78	
